# Comparative Analysis of Knee Biomechanics in Total Knee Arthroplasty Patients Across Daily Activities

**DOI:** 10.3390/bioengineering12101018

**Published:** 2025-09-25

**Authors:** Fangjian Chen, Hannah Seymour, Naiquan (Nigel) Zheng

**Affiliations:** Department of Mechanical Engineering and Engineering Science, University of North Carolina at Charlotte, 9201 University City Blvd., Charlotte, NC 28223, USA; fchen6@charlotte.edu (F.C.); stokes.hannah@gmail.com (H.S.)

**Keywords:** knee biomechanics index, motion analysis, stair ambulation, gait analysis

## Abstract

Total knee arthroplasty (TKA) is a commonly conducted surgery to relieve pain and enhance mobility in patients with end-stage knee osteoarthritis. Patient-reported outcome measures are often used whereas biomechanical variables are too complicated for clinicians and patients to assess functional improvement. There is a need for a simplified integrated knee biomechanics index (KBI) to compare improvements in TKA patients across various daily activities and examine the relationships between clinical functional tests and daily activities. Age-, gender-, and BMI-matched three groups (20 each in posterior stabilized TKA, bi-cruciate stabilized TKA, and healthy controls) were recruited and tested pre-op and 6-month post-op to perform walking on level, slope, and stairs, and two clinical tests (timed-up-go, 10-time sit-to-stand). Knee joint kinematics and kinetics variables were calculated from motion data and ground reactions captured at 120 Hz and 1200 Hz, respectively. KBI was developed based on these variables relative to healthy controls. The longitude comparison of KBI and the differences of KBI across various daily activities were identified using repeated-measure ANOVA. Pearson correlation analysis was used to compare clinical tests and KBI of daily activities. KBIs of five daily activities were significantly increased following TKA follow-up. KBI improvement during level walking was significantly higher than those during stair ascending and descending. Significant correlations were found between timed-up-go test time and KBIs for stair ascending and descending.

## 1. Introduction

Knee osteoarthritis (OA) was one of the most common causes of disability and has increased steadily in the population over 65 years. A recent study found that OA has started to impact people under 60 years [1]. As a surgical intervention to treat end-stage knee OA, total knee arthroplasty (TKA) was a common effective orthopedic surgery well-widely performed across the United States [2,3]. While most individuals who undergo TKA report functional improvements [4], pain relief [5], and satisfaction with their surgical outcomes [6], there is still a noticeable difference between TKA patients and healthy controls in terms of objective clinical assessment [7].

In addition to pain reduction, improvements in the ability to perform daily activities are essential for TKA patients and can significantly impact their quality of life. Although previous studies have found that TKA patients restore their knee range of motion during level walking within six months of the surgery [8], deficits in sagittal plane knee moment and angle have been reported compared to the control group [9,10,11]. Walking on a slope (ramp up and ramp down) is a challenging task compared to level walking in daily activities [12,13,14,15]. It is reported that TKA patients generate higher knee abduction moments, and greater knee extension moments and quadriceps muscle activities than level walking [16,17]. Stair ambulation (ascending and descending) is considered a high-demanding daily activity requiring greater knee flexions and synergy of low extremities [18,19]. Researchers reported that the post-operative TKA patients still had significant deficits in the front plane moment and sagittal plane angle compared to healthy controls during stair ambulation [11,20,21].

Due to the complexity of biomechanics variables—such as three-dimensional kinetics and kinematics, along with bilateral differences—it becomes challenging for clinicians and researchers to fully grasp and make informed decisions about the functional performance of TKA during daily activities. To address this, a simplified integrated variable, the knee biomechanics index (KBI), was developed based on biomechanical variables with respect to healthy controls and used as the primary variable to explore the longitudinal differences (pre-op vs. post-op) and the horizontal comparison of different daily activities. Previously, we calculated the KBI using the means and standard deviations of healthy controls, which could assign different KBI values to a biomechanical variable near a borderline [22]. We have now revised the calculation method by using probabilities derived from a normal distribution curve based on Z-scores.

The aim of this study is twofold: (1) to compare KBI improvements in TKA patients across various daily activities, including high-demanding activities following TKA, and (2) to examine the relationship between commonly used clinical functional tests and KBIs of five daily activities. We hypothesized that (**H1.**) there were no significant differences in KBIs of TKA patients among different daily activities, (**H2.**) and there were no significant differences in KBIs between pre-op and post-op during all daily activities, and (**H3.**) there were no significant correlations between objective clinical assessments and KBIs of five daily activities.

## 2. Materials and Methods

### 2.1. Subjects

A total of 52 participants with unilateral TKA who underwent either posterior stabilized or bi-cruciate stabilized TKA were recruited from a local orthopedic clinical center (OrthoCarolina, Charlotte, NC, USA). The study was approved by an institutional review board (UNC Charlotte, IRB 17-0300), and all participants provided written informed consent and received permission from their surgeons to perform daily activities. Participants were excluded if they had (1) low back pain or any diseases in other ankle or hip joints, (2) a BMI greater than 38, or (3) uncontrolled blood pressure, diabetes mellitus, neoplasms, or neurological disorders such as Parkinson’s disease, impaired sensation, or stroke.

All participants between the ages of 52 and 75 were scheduled to participate in the study before surgery and were expected to return for testing six months after surgery. Unfortunately, eight participants were unable to return for testing due to unexpected reasons such as changing their surgery type, moving out of town, or having bilateral knee surgery. The average time for post-surgery return was nine months. Data from 20 participants with posterior stabilized TKA and 20 with bi-cruciate stabilized TKA, and 20 age-, gender-, and BMI-matched participants with no reported musculoskeletal injury as a control group were used for analysis (Table 1).

### 2.2. Experimental Protocols

A 10-camera motion capture system (Vicon, Oxford, UK) was used to capture the three-dimensional motion of the reflective markers at 120 Hz. A total of 52 reflective markers were placed bilaterally on bone landmarks and body segments, which included anterior superior iliac spines, posterior superior iliac spines, sacrum, medial, and lateral femoral epicondyles, medial and lateral ridges of the tibial plateau, the medial and lateral malleoli and the second metatarsal head and the heel, and anterolateral side of the thigh and the shank, as previously reported [22]. An elastic wrap was affixed to the waist to minimize the movement of artifacts. The study commenced with participants completing general medical history and demographic forms. Subsequently, they filled out the Knee Society Score Form and the Short Form Survey-12 at four intervals: pre-operation, 1 month post-operation, 6 months post-operation, and 1 year post-operation. The Forgotten Joint Score Form was administered at 1 month, 6 months, and 1 year post-operation. Findings regarding patient-reported outcomes measures have been reported previously [22].

**Figure 1:**
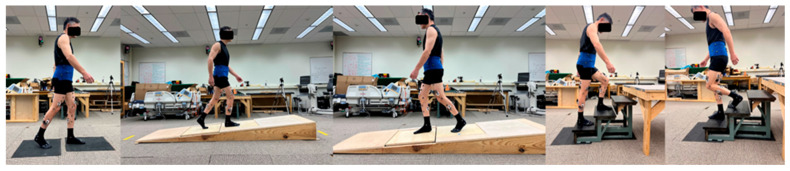
A participant performing motion tasks (left to right: level walking (LW), ramp up (RU), ramp down (RD), stair ascending (SA), stair descending (SD)). The participants consented to having these images published.

In addition, two floor-embedded force platforms (AMTI, Watertown, MA, USA) synchronized with the motion capture system were used to measure ground reaction forces and moments at 1200 Hz. T-pose was first captured which required participants with two feet parallel and arms raised to shoulder height. Two clinical assessments, timed-up-go (TUG) and ten-time sit-to-stand (STS), and five daily activities at self-selected speed level were captured. These five tasks, level walking (LW), ramp up (RU), ramp down (RD), stair ascending (SA), and stair descending (SD), were selected because they represent common and functionally critical activities of daily living that challenge the knee joint in different ways. Level walking is the most frequent locomotor activity and serves as a baseline for gait analysis. Ramp walking (both ascent and descent) introduces altered loading conditions and requires increased quadriceps and hip extensor activity, making it relevant for assessing functional adaptation to inclined surfaces encountered in real-world environments. Stair ambulation is widely recognized as one of the most demanding daily tasks for individuals with TKA, requiring greater knee flexion, higher joint moments, and coordinated muscle activation compared to level walking. Including these tasks allows for a comprehensive assessment of knee biomechanics across a spectrum of low- to high-demand activities, which is essential for understanding residual limitations and guiding rehabilitation strategies. A customized ramp (3 m long, 1 m wide, and 15 degrees) and stairs (4 steps, 18 cm high, and 45 cm wide) were used to simulate common, daily life activities. Wood plates were placed above the force plates during ramp walking to capture ground reaction force, and the steps contacting each force plate during stair ambulation ensured that the ground reaction force of one foot was captured by one force plate [22,23,24] (Figure 1).

All tasks were performed at a self-selected comfortable speed to preserve natural gait patterns (consistent with prior TKA gait protocols [8,10,11]). For each activity, five successful trials were collected. Three valid trials were processed, and their ensemble average (per limb, per activity) was used to represent each participant. “Failure” was operationally defined as the inability to safely initiate or complete a task (e.g., loss of balance or need for assistance). Trials aborted for technical reasons (e.g., marker occlusion or force-plate targeting) were repeated and were not coded as failures; only safety-terminated attempts received a zero KBI for that activity.

### 2.3. Motion Data Analysis

The motion trajectories were filtered with a low-pass filter at 6 Hz to remove high-frequency noise, while ground reaction force and moment data were filtered with a cut-off frequency of 15 Hz. Joint kinematics were derived from the motion of lower body segments, including the foot, tibia, femur, and pelvic regions, with the defined local coordinates system. The joint centers of the ankle, knee, and hip were predicted with the mid-point between the lateral and medial malleoli, between the lateral and medial femoral epicondyles, and markers on the pelvic region [25,26]. The motion of each segment during daily activities was then calculated using a least mean square-based algorithm [27,28]. Three-dimensional joint angles (flexion/extension, varus/valgus, and internal/external rotation) were calculated using the projection method [29]. Three-dimensional joint moments (flexion/extension, abduction/adduction, and external/internal) were derived using an inverse dynamics model and normalized to the product of height and body weight (%BW*H). The initial contact (foot strike) and toe-off were detected using a threshold of 5% body weight of ground reaction force to determine the stance and swing phases of joint kinematic and kinetic patterns. A gait cycle from both limbs of each trial was used for analysis, and time was normalized to 100% of the step cycle [23].

### 2.4. Knee Biomechanics Index

To better understand the differences in biomechanics during five distinct daily activities, KBI was developed based on knee kinetics and kinematics. Knee kinematics were analyzed across three different planes of motion: sagittal, frontal, and transverse. The sagittal plane (flexion–extension) range of motion (FERoM) was defined as the difference between the peak and valley flexion joint angles during the gait cycle. The frontal plane (varus–valgus) range of motion (VVRoM) was defined as the difference between the peak and valley varus joint angles during the gait cycle. The transverse plane (internal–external) range of motion (INTRoM) was defined as the difference between the peak and valley internal rotation joint angles during the gait cycle. Knee kinetics were analyzed in three different planes as well, including the peak and valley sagittal plane (flexion–extension) moment (FEM), the peak and valley front plane (abduction–adduction) moment (AAM), the peak and valley transverse plane (internal–external) moment (IEM), and knee contact force (KCF) during the gait cycle.

Several previous studies have utilized variables such as sagittal plane range of motion [30,31,32,33,34], sagittal plane moment [26,35,36,37], and the bilateral difference of the ground reaction forces [38,39] as main variables to compare the TKA patients with control group during level walking and stair ambulation. These variables are considered crucial for several reasons: optimizing knee flexion and extension moments enhances muscle strength and balance around the knee joint; improving these moments, along with knee flexion and extension range of motion, can reduce joint stress, thereby alleviating pain in patients with knee osteoarthritis and TKA; and bilateral differences can lead to increased stress and uneven weight distribution on the contralateral knee, thereby raising the risk of bilateral TKA [40]. Consequently, knee flexion and extension range of motion and moments, as well as the bilateral ratio of knee contact force (KCF), are critical for functional improvements in patients with knee osteoarthritis and total knee arthroplasty.

Additionally, studies on TKA have also examined knee kinematics and kinetics in the frontal and transverse planes. For instance, the maximum external knee adduction moment was found to be lower in TKA patients [32,35]. Contradictorily, Standifird et al. [36] reported an increased peak knee abduction moment compared to controls, while Saari et al. [41] reported no difference between TKA patients and control subjects in knee abduction moments. Reduced knee abduction and adduction moments can alleviate medial or lateral forces on the knee, potentially decreasing the risk of excessive joint loading and degeneration [11]. Additionally, improvements in transverse plane moments and rotations can be indicative of functional improvements in TKA patients. However, these are not the sole factors to consider when evaluating overall functional improvements.

**Figure 2:**
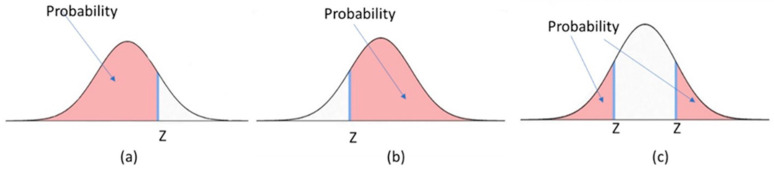
The calculation method of probabilities of the normal distribution curve with a given Z-score. (**a**) Probability calculated as higher values are preferable; (**b**) Probability calculated as lower values are preferable; and (**c**) Probability calculated as the value of one is ideal for variables like bilateral ratio.

All variables from the pre-op and post-op groups were compared to those of the control group. Z-scores were calculated based on the mean and standard deviation of the control group, serving as a threshold. Participants were then assigned probabilities based on a normal distribution curve using these Z-scores. For variables like FERoM, INTRoM, FEM, and IEM, where higher values are preferable, probabilities were calculated as shown in Figure 2a. For variables like AAM, where lower values are preferable, probabilities were calculated as shown in Figure 2b. For variables like the bilateral ratio of KCF, where the value of one is ideal, probabilities were calculated as shown in Figure 2c. Participants who failed to perform a specific motor task received zero points. The KBI was then calculated by summing the probabilities for these six variables. The selection of these six variables was based on prior literature identifying their clinical relevance, rather than on statistical reduction techniques such as factor analysis. All six variables contributed equally to the composite KBI (unweighted sum of probabilities). Equal weighting was chosen to maximize interpretability and clinical usability. A perfect KBI score is six. The KBI scores for the pre-operative, post-operative, and control groups were determined based on the mean and standard deviation of the control group.

### 2.5. Statistical Analysis

A one-way analysis of variance (ANOVA) was used to identify demographic differences between the TKA and control groups using SPSS (SPSS27; IBM). For the clinical functional test, one-way ANOVA with post hoc testing was performed in the pre-op, post-op, and control groups at a 0.05 alpha level. To examine the group (pre-op, post-op, and control group) and daily activities (LW, RU, RD, SA, and SD) interaction and main effects of knee kinematics and kinetics variables, a repeated-measure ANOVA was used. A post hoc comparison was conducted to identify differences between the pre-op, post-op, and control groups. To investigate differences in the KBI among participants in the pre-operative, post-operative, and control groups, an ANOVA followed by a post hoc test was employed. This analysis aimed to identify differences in TKA follow-up during daily activities among the groups. To explore variations in daily activities, repeated-measures ANOVA was conducted, with the groups (pre-op and post-op) and tasks (five different daily activities) as factors, to evaluate the KBI. Additionally, Pearson’s correlation analysis was used to compare the functional tests and KBI measurements across the five daily activities. Gait speed was recorded for all trials; because our objective was to compare ecologically valid performance at each participant’s comfortable speed, gait speed was not included as a covariate in the primary repeated-measures models.

## 3. Results

No significant differences in age, height, or BMI were found between the TKA and control groups (Table 1). While there were no significant differences in TUG performance time among the pre-op, post-op, and control groups, the control and post-op groups performed TUG 20% faster than the pre-op group. Significant differences were observed in STS performance time among the pre-op, post-op, and control groups. Post hoc testing revealed that the control group performed STS significantly faster than the pre-op and post-op groups, with the post-op group performing STS 15% faster than the pre-op group (Table 2).

A significant group × task interaction was found in FERoM (*p* < 0.01, Table 3), with significant task effects indicating that FERoM increased notably in SA and SD compared to the other three activities. Post hoc comparisons revealed that FERoM in the post-op group was significantly greater than that in the pre-op group, while both groups had smaller FERoM values than the control group. The post-op group had significantly greater INTRoM than the pre-op group, and INTRoM in both groups was significantly lower than in the control group. No significant group × task interaction or main effects were observed in VVRoM.

A significant group × task interaction was observed in FEM (*p* < 0.01, Table 4), with a significant task main effect indicating that FEM increased as the difficulty of daily activities increased. Post hoc comparisons revealed that FEM in the post-op group was significantly greater than in the pre-op group, and FEM values in both groups were significantly lower than in the control group. The post-op group had significantly lower AAM than the pre-op group, and AAM values in both groups were significantly higher than those in the control group. For IEM, no significant group × task interaction or main effect was found. A significant group × task interaction was detected in the bilateral ratio of KCF (*p* < 0.01, Table 4), with a significant task main effect. Post hoc comparisons indicated that the bilateral ratio of KCF in the post-op group was significantly greater than in the pre-op group, and the bilateral ratio of KCF in both groups was significantly lower than in the control group.

**Figure 3:**
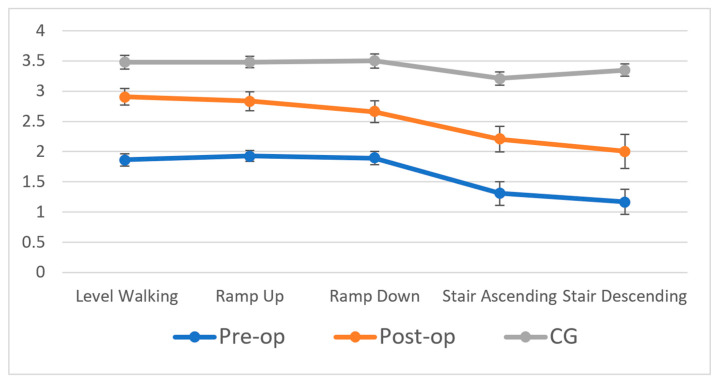
Knee biomechanical index in five daily activities for TKA follow-up and control group.

In the follow-up comparisons of TKA patients, significant differences were observed among the pre-operative, post-operative, and control groups during various activities: level walking, ramp up, ramp down, stair ascending, and stair descending (Figure 3). Post hoc analysis revealed that TKA patients experienced significant improvements in KBI. Specifically, there was a 61% increase during level walking, a 47% increase during ramp up, a 42% increase during ramp down, a 69% increase during stair ascending, and a 67% increase during stair descending when compared to pre-op measures (Table 5). Unfortunately, even after surgery, the post-op group’s KBI values still lagged those of the control group. The control group exhibited a 20% higher KBI during level walking, 25% higher during ramp up, 30% higher during ramp down, 45% higher during stair ascending, and 65% higher during stair descending, as compared to the post-op group.

For the comparison with five different activities, significant main effect for the group was detected in KBI, showing that KBI scores for five daily activities significantly increased at follow-up. Post hoc analysis revealed that the KBI for level walking was significantly higher than those for stair ascending and stair descending. There were no significant differences in KBIs between the KBI scores for level walking and ramp up, or between those for level walking and ramp down. Similarly, the KBI for ramp up was significantly higher than for stair ascending and stair descending but showed no significant difference compared to level walking and ramp down. As for ramp descending, the KBI was also significantly higher than for stair ascending and stair descending, and it showed no significant difference compared to level walking and ramp up. In terms of KBI improvements, the extent of enhancement in daily activities decreased as the difficulty of the activity increased. Specifically, the improvements in KBI for level walking were significantly greater than those for stair ascending and stair descending.

In the pre-op group, significant correlations were observed between the TUG performance time and KBI scores for ramp down, stair ascending, and stair descending. However, no significant correlations were found between the STS performance time and KBI scores across the five daily activities, nor were any found between gait speed and KBI scores for these activities (*p* > 0.05 for all comparisons; Table 6). In the post-op group, significant correlations were noted between TUG performance time and KBI scores for ramp up, ramp down, stair ascending, and stair descending. Additionally, significant correlations were observed between STS performance time and KBI scores for level walking, stair ascending, and stair descending. However, no significant correlations were detected between gait speed and KBI scores for any of the daily activities.

## 4. Discussion

It is demonstrated that a knee biomechanics index derived from six major biomechanical variables of interest can be used to compare the performance of individuals with TKA during five daily activities. The comparison of pre-op and post-op index scores reveals improvements in performance during daily activities after TKA follow-up. Additionally, correlations between clinical function tests and KBIs of daily activities were developed, which can assist surgeons in determining the rehabilitation needs of TKA patients based on these clinical tests.

Our first hypothesis was rejected as the knee biomechanics index of stair ascending and stair descending were significantly lower than those of ramp up, ramp down, and level walking. In terms of the flexion–extension range of motion (FEROM), we found that during stair ascending and stair descending, pre-op measures were 51% and 46.7% higher compared to level walking, and 50.2% and 43.6% higher in the post-op stage, respectively. Additionally, the flexion–extension moment (FEM) in level walking was 54.2% and 63.1% lower than in stair ascending and stair descending at both pre- and post-op stages. These findings of sagittal plane knee kinematics and kinetics indicate that individuals with TKAs performing stair ambulation require greater functional capabilities. Previous studies have also reported differences between walking and stair ambulation [20,42,43,44] and between level walking and ramp walking [14,17,45,46,47]. Although no significant difference was observed in KBI between level and inclined walking, it is worth noting that the KBI is derived from six main biomechanical variables, providing a more comprehensive understanding of daily activities. Additionally, we observed a tendency for the KBI to decrease as activities transitioned from level walking to stair descending.

This data suggests that even at nine months post-op, individuals with TKA continue to face difficulties in performing stair ambulation when compared to level walking, ramp up, and ramp down. These functional deficits may be attributable to incomplete recovery of lower extremity muscles and restricted knee joint range of motion. Such findings could aid clinicians in defining more effective rehabilitation criteria and improving rehab protocols for TKA patients.

The second hypothesis was rejected, given the significant differences observed between pre-op and post-op in KBIs during five daily activities. According to the ANOVA with post hoc testing, post-op scores showed a 61% increase in level walking, a 47% increase in ramp up, a 42% increase in ramp down, a 69% increase in stair ascending, and a 67% increase in stair descending compared to pre-op measures. For the three-dimensional knee kinematics, both FERoM and INTRoM showed significant differences with TKA follow-up. Compared to the pre-op, the FEM was significantly greater at the post-op, while the AAM showed significantly lower during daily activities (Table 3 and Table 4). The previous studies have reported similar results [11,20]. The increased FEM in the post-op group may be attributed to the increased FERoM and the recovery of lower extremities’ strength. The significant decrease in AAM indicates that the knee stability of the front plane was improved. These findings suggest that individuals with TKA experienced functional improvements and increased knee joint stability at follow-up. Despite these functional gains, post-op individuals still exhibited deficits when compared to the healthy controls. Specifically, the KBI scores in the control group were 45% higher during stair ascending and 65% higher during stair descending. Furthermore, the control group’s FERoM, INTRoM, and FEM measures were significantly higher, consistent with previously reported studies [21,48,49,50]. Although TKA participants showed significant improvements across all daily activities, deficits were more pronounced in high-demand activities like stair ambulation. This suggests that stair ambulation remains a challenging activity for TKA patients post-operatively and that the time required to reach the functional level of the healthy controls is longer than expected.

The third hypothesis is partly supported by the correlation found between the time taken to perform functional tests and the KBIs during daily activities. Clinical functional tests have been found to be useful for assessing TKA patients with limited mobility and for predicting their functional performance [48,51]. Previous studies have reported negative correlations between clinical functional assessments and muscle strength, suggesting that higher muscle strength is necessary for faster performance times. The lack of significant differences found in the pre-op group may be due to the inconsistent conditions of the involved limb, which can cause performance variations across different activities. Nonetheless, our study found negative correlations between ramp down and TUG, stair ascending and TUG, as well as stair descending and TUG, in both the pre-op and post-op groups. This may suggest that the TUG functional test behaves similarly across daily activities. Moreover, the strong correlation observed during high-demand activities indicates that KBI scores may be related to clinical functional test outcomes. On the other hand, since the STS test requires higher low extremities muscle strength compared to the TUG, significant correlations were only detected between stair ascending and STS and between stair descending and STS. These findings imply that clinical functional tests can be effective predictors of functional performance in individuals with TKA, particularly in high-demand activities.

The KBI offers a comprehensive insight into the functional performance of the knee during daily activities. This is achieved by integration of the six most important biomechanical variables. Such integration allows for clear differentiation between the performance of participants during high-demand and low-demand activities. Additionally, the KBI visibly displays functional improvements in TKA follow-ups, highlighting deficits when compared to healthy controls. The accuracy of the KBI, which is determined based on the normal distribution of healthy controls, hinges upon the sampling size and composition of these controls. More convincing results can be obtained if the data is comprehensive, especially when matched with age, gender, and BMI. The KBI is intended as an adjunct rather than a replacement for detailed biomechanical evaluation. Its purpose is to provide clinicians with a simplified, integrated measure of knee function that complements, rather than substitutes, the granular analysis of individual kinematic and kinetic variables. While the composite score facilitates quick interpretation and longitudinal tracking, the underlying variables remain fully accessible for targeted treatment planning.

In future applications, the KBI might serve as a tool to contrast differences between two distinct knee implants. When data from an entire population with a specific knee implant is available, an individual’s KBI can be juxtaposed with the collective data of the same implant users. By comparing KBI scores in this group, individuals can discern their ranking among peers with the same knee implant, age, and gender, and this may motivate TKA patients to engage more actively in rehabilitation to achieve a higher ranking.

While we describe lower post-operative KBI values relative to controls as ‘deficits,’ some observed patterns may reflect protective or compensatory adaptations aimed at reducing joint loading or enhancing stability. For example, lower sagittal plane moments and persistent asymmetries during stair negotiation have been linked to altered loading strategies in TKA populations [19,26]. Determining whether such adaptations are beneficial or detrimental over the long term will require longitudinal follow-up.

The present KBI was derived from laboratory motion capture and force-plate data, which may limit immediate clinical deployment. To bridge this gap, future work will validate simplified KBI estimators using wearable inertial measurement units and pressure-sensing insoles to approximate kinematics, moments, and load symmetry, as well as markerless video-based motion analysis. These approaches would enable point-of-care assessments while benchmarking against the gold-standard laboratory system. Additionally, habitual activity levels were not controlled between groups, which may influence gait mechanics; future studies will include validated activity measures to match or adjust for activity. The normative reference cohort included 20 healthy controls; although consistent with prior gait studies, expanding this dataset across age, sex, and BMI strata will improve the precision of Z-score-based probabilities. To validate the assumption of normality in the control group, we performed Shapiro–Wilk tests on all primary kinematic and kinetic variables, which indicated no significant deviations from normality, supporting the use of parametric analyses. While we used self-selected speeds to preserve ecological validity, gait speed can influence kinetics and kinematics; speed-matched subsets and covariate adjustments will be examined in future analyses. Finally, the current KBI uses equal weighting across variables; we will perform sensitivity analyses of alternative weighting schemes to quantify robustness.

The decision to adopt a small, well-matched control group (*n* = 20) to establish normative thresholds is grounded in both precedent and statistical validity. Prior biomechanical studies in TKA have successfully employed similar sample sizes to derive meaningful insights. For instance, Catani et al. analyzed in vivo kinematics using 20 subjects [8], while Mandeville et al. and Standifird et al. conducted gait and stair ascent analyses with cohorts of 15–18 participants [11,36]. These studies aimed to quantify joint mechanics during functional tasks, compare TKA patients with healthy controls, and identify compensatory movement patterns—objectives that closely align with the present work.

Our study mirrors these designs in several key respects. The control group was matched to the TKA cohort by age, gender, and BMI, minimizing confounding variables and enhancing the precision of normative comparisons. Measurement tools such as a 10-camera Vicon motion capture system and synchronized force plates were used, consistent with industry standards. Furthermore, the outcome variables selected—FERoM, INTRoM, FEM, AAM, IEM, and KCF—are well-established indicators of joint function and have been utilized in the studies [8,11,36].

Statistically, the use of a small sample size in exploratory normative research is valid under specific conditions. We confirmed normal distribution of control data using Shapiro–Wilk tests, which supports the use of parametric analyses and Z-score modeling. Hatfield et al. demonstrated that control groups of 68 participants can reliably establish biomechanical baselines [33], while Fantozzi et al. showed that as few as 20 TKA participants can reliably detect differences between two implants [35], particularly when effect sizes are large and measurement reliability is high.

We acknowledge that the absence of a single, large-scale, demographically representative normative dataset remains a limitation. Nevertheless, by integrating Z-scores with probability curves, our approach enables nuanced interpretation of individual deviations from normative function, even within a modest dataset.

Several other limitations of this study warrant consideration. First, the analysis focused exclusively on knee kinematics and kinetics, neglecting the potential contributions and compensations of hip and ankle variables during high-demand activities. Second, the study involved only posterior stabilized and bi-cruciate stabilized TKA implants, leaving the performance of cruciate retaining implants during daily activities unexplored. Future research should gather more data to compare these implant types. Third, the investigation was limited to walking on a fixed incline, potentially constraining the applicability of the findings to other ramp walking situations. Finally, the stair ambulation test required participants to place one foot on each step, potentially excluding individuals who were unable to execute the task correctly.

## 5. Conclusions

It is concluded that TKA surgery provided functional improvements for patients during daily activities, but they might still experience deficits when performing high-demanding activities such as stair ambulation. The proposed knee biomechanics index is a valid and simplified tool to integrate all important biomechanical variables of the knee joint for clinicians. Clinical functional tests can be helpful in predicting the functional improvements of daily activities for TKA patients.

## Figures and Tables

**Table 1:** Descriptive data for participants in TKA group and control group (mean ± standard deviation). The *p*-value is a statistical measure used to compare differences among the three groups.

	Posterior Stabilized	Bi-Cruciate Stabilized	Control Group	*p*-Value
Age (years)	64.9 ± 5.8	65.4 ± 6.5	63.4 ± 7.5	0.357
Gender (M/F)	11/9	11/9	11/9	NA
Height (m)	1.72 ± 0.10	1.70 ± 0.10	1.70 ± 0.10	0.481
BMI (kg/m^2^)	30.7 ± 4.6	30.6 ± 4.3	27.1 ± 4.4	0.15

**Table 2:** Clinical functional tests (TUG: timed-up-go, STS: ten-time sit-to-stand) (mean ± standard deviation).

	Pre-Op	Post-Op	Control Group
TUG(s)	11.8 ± 6.4	9.9 ± 2.5	9.0 ± 1.9
STS(s)	33.9 ± 10.2 ^ab^	28.9 ± 8.6 ^a^	25.45 ± 4.8 ^b^

^a^: Significant difference between pre and post; ^b^: significant difference between pre and control group.

**Table 3:** Knee kinematics variables (FEROM: flexion–extension range of motion, VVRoM: varus–valgus range of motion, INTRoM: internal–external range of motion) (mean ± standard deviation).

Variable	Group	LW	RU	RD	SA	SD
FERoM *^^abc^ (degree)	Pre-op	49.07 ± 6.37	44.58 ± 6.61	49.35 ± 7.37	74.09 ± 5.19	72.04 ± 7.25
	Post-op	56.05 ± 6.64	50.9 ± 7.02	55.48 ± 6.81	84.2 ± 6.55	80.46 ± 5.7
	CG	59.59 ± 3.53	55.34 ± 5.03	59.16 ± 5.57	91.97 ± 4.78	88.35 ± 6.21
VVRoM (degree)	Pre-op	9.7 ± 2.65	9.08 ± 3.16	9.47 ± 3.56	10.65 ± 3.6	9.75 ± 3.36
	Post-op	10.11 ± 4.58	11.87 ± 8.72	11.14 ± 9.02	14.06 ± 5.64	12.22 ± 4.41
	CG	12.35 ± 5.72	11.08 ± 4.28	13.43 ± 5.5	11.51 ± 4.08	11.33 ± 5.15
INTRoM ^abc^ (degree)	Pre-op	14.53 ± 5.21	14.26 ± 4.66	15.21 ± 4.97	13.22 ± 4.38	14.12 ± 4.24
	Post-op	17.66 ± 5.08	17.11 ± 5.55	16.57 ± 4.92	18.2 ± 5.45	19.08 ± 6.16
	CG	18.86 ± 6.22	18.91 ± 5.19	19.99 ± 6.52	22.11 ± 7.6	21.82 ± 6.04

* Significant task (daily activities) main effect; ^^^ significant interaction group task interaction; ^a^: significant difference between pre-op and post-op; ^b^: significant difference between pre-op and control group; ^c^: significant difference between post-op and control group.

**Table 4:** Knee kinetics variables (FEM: flexion–extension moment, IEM: internal–external moment, AAM: abduction–adduction moment, KCF: knee contact force) (mean ± standard deviation) (%BWxH).

Variable	Group	LW	RU	RD	SA	SD
FEM *^^abc^ (%BWxH)	Pre-op	2.68 ± 0.95	2.83 ± 1.23	3.14 ± 1.22	5.83 ± 1.60	7.65 ± 2.24
	Post-op	3.70 ± 1.16	3.36 ± 0.91	4.51 ± 1.33	7.90 ± 2.40	8.89 ± 2.78
	CG	4.43 ± 1.11	4.58 ± 1.46	5.29 ± 1.27	9.21 ± 1.93	9.37 ± 1.78
IEM (%BWxH)	Pre-op	0.78 ± 0.46	0.78 ± 0.47	0.74 ± 0.40	0.92 ± 0.88	0.88 ± 0.64
	Post-op	0.82 ± 0.33	0.73 ± 0.32	0.75 ± 0.51	0.90 ± 0.73	0.84 ± 0.61
	CG	0.60 ± 0.27	0.47 ± 0.29	0.59 ± 0.28	0.57 ± 0.52	0.45 ± 0.31
AAM *^abc^ (%BWxH)	Pre-op	2.95 ± 1.35	3.02 ± 1.39	3.10 ± 1.34	3.81 ± 0.66	3.81 ± 0.73
	Post-op	2.58 ± 0.89	2.16 ± 0.88	2.41 ± 1.19	3.51 ± 0.63	3.64 ± 1.13
	CG	1.62 ± 0.62	1.53 ± 0.71	1.87 ± 0.88	2.85 ± 0.24	2.87 ± 0.36
Bilateral ratio of KCF *^^abc^	Pre-op	0.82 ± 0.03	0.82 ± 0.04	0.8 ± 0.04	0.78 ± 0.07	0.77 ± 0.08
	Post-op	0.9 ± 0.04	0.88 ± 0.06	0.85 ± 0.08	0.84 ± 0.06	0.83 ± 0.08
	CG	1.02 ± 0.04	1.03 ± 0.03	1.00 ± 0.05	1.03 ± 0.08	0.95 ± 0.07

* Significant task (daily activities) main effect; ^^^ significant interaction group task interaction; ^a^: significant difference between pre-op and post-op; ^b^: significant difference between pre-op and control group; ^c^: significant difference between post-op and control group.

**Table 5:** Knee biomechanics index (KBI) (mean ± standard deviation) for participants during daily activities (LW: level walking, RU: ramp up, RD: ramp down, SA: stair ascending, SD: stair descending).

KBI *^^^	Pre-Op	Post-Op	CG
Level Walking ^cd^	1.86 ± 0.2	2.91 ± 0.28	3.48 ± 0.23
Ramp Up ^fg^	1.93 ± 0.18	2.84 ± 0.31	3.48 ± 0.19
Ramp Down ^ik^	1.89 ± 0.22	2.66 ± 0.37	3.5 ± 0.23
Stair Ascending	1.31 ± 0.39	2.21 ± 0.43	3.21 ± 0.22
Stair Descending	1.17 ± 0.42	2.01 ± 0.57	3.35 ± 0.2

* Significant group main effect; ^^^ significant interaction group task interaction; ^c^: significant difference between Level Walking and Stair Ascending; ^d^: significant difference between Level Walking and Stair Descending; ^f^: significant difference between Ramp Up and Stair Ascending; ^g^: significant difference between Ramp Up and Stair Descending; ^i^: significant difference between Ramp Down and Stair Descending; ^k^: significant difference between Ramp Down and Stair Descending.

**Table 6:** Correlation between KBI during daily activities and clinical functional tests (* *p* < 0.05; ** *p* ≤ 0.01).

Activity	Pre–Post	TUG		STS		Gait Speed	
		r	*p*	r	*p*	r	*p*
Level walking	pre	0.12	0.46	0.05	0.76	−0.06	0.71
	post	−0.23	0.15	−0.32	0.04 *	0.24	0.13
Ramping up	pre	0.20	0.22	0.05	0.76	−0.27	0.09
	post	−0.41	0.01 **	−0.23	0.15	0.16	0.32
Ramping down	pre	0.35	0.03 *	0.03	0.85	−0.12	0.46
	post	−0.38	0.02 *	−0.27	0.09	0.27	0.09
Stair ascending	pre	−0.33	0.04 *	−0.11	0.50	−0.14	0.39
	post	−0.41	0.01 **	−0.50	0 **	0.11	0.50
Stair descending	pre	−0.32	0.04 *	−0.17	0.29	0.11	0.50
	post	−0.45	0 **	−0.50	0 **	0.13	0.42

## Data Availability

The data presented in this study are available on request from the corresponding author due to privacy or ethical restrictions.

## Abbreviations

The following abbreviations are used in this manuscript:

OA	Osteoarthritis
KBI	Knee Biomechanics Index
TKA	Total knee arthroplasty
TUG	Time up and go
STS	Sit-to-stand
LW	level walking
RU	ramp up
RD	ramp down
SA	stair ascending
SD	stair descending
FERoM	flexion–extension range of motion
VVRoM	varus–valgus range of motion
INTRoM	internal–external rotation range of motion
FEM	flexion–extension moment
AAM	abduction–adduction moment
IEM	internal–external rotation moment
KCF	knee contact force
